# A Longitudinal Framework to Describe the Relation Between Age-Related Hearing Loss and Social Isolation

**DOI:** 10.1177/23312165241236041

**Published:** 2024-03-28

**Authors:** Aysha Motala, Ingrid S. Johnsrude, Björn Herrmann

**Affiliations:** 1 Department of Psychology & Brain and Mind Institute, 6221 University of Western Ontario, London, Canada; 2 Department of Psychology, 7622 University of Stirling, Scotland, UK; 3 School of Communication Sciences & Disorders, 6221 University of Western Ontario, London, Canada; 4 63670 Rotman Research Institute, Baycrest Hospital, Toronto, Canada; 5 Department of Psychology, University of Toronto, Toronto, Canada

**Keywords:** hearing loss, social isolation, aging, speech understanding, communication, auditory perception

## Abstract

Many older adults live with some form of hearing loss and have difficulty understanding speech in the presence of background sound. Experiences resulting from such difficulties include increased listening effort and fatigue. Social interactions may become less appealing in the context of such experiences, and age-related hearing loss is associated with an increased risk of social isolation and associated negative psychosocial health outcomes. However, the precise relationship between age-related hearing loss and social isolation is not well described. Here, we review the literature and synthesize existing work from different domains to propose a framework with three conceptual anchor stages to describe the relation between hearing loss and social isolation: within-situation disengagement from listening, social withdrawal, and social isolation. We describe the distinct characteristics of each stage and suggest potential interventions to mitigate negative impacts of hearing loss on social lives and health. We close by outlining potential implications for researchers and clinicians.

## Introduction

The World Health Organization's World Report on Hearing (2021) indicates that over 65% of adults aged 60 years or above experience some form of hearing loss (Cruickshanks et al., 1998; Feder et al., 2015; Helfer et al., 2017), such as speech understanding difficulties when background sound is present (Pang et al., 2019; Schneider et al., 2000). Speech masked by background sound requires a listener to invest cognitively to understand what is said (Hicks & Tharpe, 2002; Van Engen et al., 2012), and this cognitive load appears to be particularly high for older adults with hearing loss (Pichora-Fuller et al., 2016; Rönnberg et al., 2013). Common aversive experiences that result from such comprehension difficulties and cognitive investment include feelings of listening effort, fatigue, and frustration (Francis & Oliver, 2018; Herrmann & Johnsrude, 2020; Hornsby et al., 2016; McGarrigle et al., 2014; Pichora-Fuller et al., 2016) that make social interactions less appealing and can lead to avoidance behaviors (Pang et al., 2019). It is thus not surprising that hearing loss has been associated with fewer social connections and an increased likelihood of social isolation (Bott & Saunders, 2021; Shukla et al., 2020).

Social isolation refers to “the absence of social interactions, contacts, and relationships with family and friends, with neighbors on an individual level, and with ‘society at large’ on a broader level” (Berg & Cassells, 1992, p. 243). Although previous work has demonstrated a correlation between age-related hearing loss and social isolation (Bott & Saunders, 2021; Cudjoe et al., 2020; Dawes et al., 2015; Shukla et al., 2020; Weinstein & Ventry, 1982), research examining the underlying drivers of this relationship is limited. This is in part because research typically focuses on later stages of age-related hearing loss after it has developed over decades (Pichora-Fuller et al., 2016; Pichora-Fuller & Levitt, 2012). However, individuals typically do not become socially isolated from one day to the next: changes to a person's social quality of life occur more gradually. Health comorbidities and other factors may also contribute to social isolation (Dawes et al., 2015), but have not been considered explicitly in the context of hearing loss. Moreover, different terms to describe social isolation, such as social withdrawal, loneliness, and social disconnectedness, have been used largely interchangeably in the literature (Ben Simon & Walker, 2018; Cornwell & Waite, 2009; Nicholson Jr, 2009; Quach & Burr, 2021), but may be dissociable in terms of their relationship with hearing loss and other factors.

Understanding the relationship between hearing loss and social isolation is not only important because social isolation is a barrier to inclusion and reduces quality of life (Weldrick & Grenier, 2018). Social isolation is also associated with cognitive decline (Biordi & Nicholson, 2013; Chern & Golub, 2019; Fratiglioni et al., 2000; Lin & Albert, 2014) and other health-related issues such as poor nutrition, smoking, alcohol abuse, depression, and heart disease (Cosh et al., 2019; Cudjoe et al., 2020; Jafari et al., 2019; Landeiro et al., 2017; Mick et al., 2018; Nicholson Jr, 2009; Pantell et al., 2013; Stam et al., 2016; Taylor et al., 2018; Wells et al., 2020). An increased risk of social isolation associated with age-related hearing loss may thus, in turn, increase the risk of an array of conditions that diminish health and well-being (Cacioppo & Cacioppo, 2014).

The current article reviews the literature on age-related hearing loss and factors that may influence the progression to social isolation, and proposes a framework that provides conceptual anchor stages that can be used to understand the relationship between hearing loss and social isolation. A more complete account of the factors that influence social behaviors, experiences, and relationships as hearing declines may help predict who is at risk of social isolation, and who will continue to participate in social interactions despite hearing challenges. We start by reviewing the literature on the decline in quality of life for older people as a result of social isolation, first broadly and subsequently in the context of hearing loss. We then describe different conceptual anchor stages associated with social isolation at which audiologists and other clinicians may be able to intervene to improve the lives of older adults. We hope that, by reviewing the literature and by identifying loci of intervention, we will help researchers and clinicians in this area develop more targeted protocols to investigate the impact of hearing loss on social isolation, and interventions that more effectively mitigate the risk of social isolation for individuals with age-related hearing loss.

## Hearing Loss in Older Adulthood

Age-related hearing loss involves changes throughout the auditory system and may include threshold elevations and/or changes in supra-threshold auditory processing (Helfer et al., 2020; Plack, 2018), resulting from damage to auditory peripheral structures such as inner and outer hair cells and the stria vascularis (Bao & Ohlemiller, 2010; Dubno et al., 2013; Gratton & Vazquez, 2003; Keithley, 2020; Moore, 2007; Plack, 2018; Schmiedt, 2010). Functional loss of synapses between inner hair cells and auditory nerve fibers (i.e., cochlear synaptopathy) may contribute to hearing impairment (Kujawa & Liberman, 2009; Liberman & Kujawa, 2017; Plack et al., 2014). Factors that likely compromise function of auditory peripheral structures in older people include long-term sound exposure from everyday activities (e.g., in cafeterias, train stations, and occupational noise), occasional events with high-intensity sound exposure (e.g., concerts, industrial noise), cardiovascular issues (e.g., high blood pressure), and treatment with medications that have ototoxic side effects (Gates & Mills, 2005; Ibrahim & Llano, 2019; Schmiedt, 2010; Zhao et al., 2016). Peripheral damage has cascading effects on afferent brain structures, leading to a loss of neural inhibition and hyperactivity in subcortical and cortical structures of the auditory system (Auerbach et al., 2014; Herrmann & Butler, 2021; Knipper et al., 2013; Resnik & Polley, 2017; Salvi et al., 2017), making hearing loss a dysfunction of the entire auditory system.

The World Report on Hearing (https://www.who.int/publications/i/item/9789240020481) refers to “normal” hearing as being able to hear a pure tone at a 20 dB hearing level (HL) or better in both ears (Chadha et al., 2021; typically in the 0.5–4 kHz frequency range), whereas an individual with a hearing threshold higher than 20 dB in this frequency range is considered to have hearing loss. Around 40% of individuals over the age of 60 years live with some type of hearing loss (Feder et al., 2015; Helfer et al., 2017; van Rooij & Plomp, 1992). Estimates of the prevalence of age-related hearing loss differ somewhat across studies (Löhler et al., 2019), but incidence and severity increase substantially with increasing age (Lin, 2011). Some studies estimate that among adults over 70 years of age, more than 66% have clinically diagnosable hearing loss (Goman & Lin, 2016). Hearing loss differs markedly between sexes (Reavis et al., 2023). It is more prevalent, more severe, and appears at an earlier age in men than in women (Feder et al., 2015; Helzner et al., 2005; Nash et al., 2011; Nolan, 2020; Pearson et al., 1995). Adults from low-income households and/or with low educational attainment are also more likely to experience hearing loss, and experience it earlier, compared to those in high-income/education households (Emmett & Francis, 2015; Feder et al., 2015; Jung & Bhattacharyya, 2012), potentially due to increased environmental and occupational noise.

Estimations of the prevalence of hearing loss, based on the current clinical criterion of elevated detection thresholds for pure tones, underestimate the true number of people with hearing loss. Many older adults experience difficulty understanding speech in the presence of background sound (Arlinger, 2003; Pichora-Fuller, 2003; Jacobson, 2018; Pienkowski, 2017; Spyridakou & Bamiou, 2015) decades before their audiometric thresholds qualify them for treatment with a hearing aid (Pichora-Fuller & Levitt, 2012). Cochlear synaptopathy in the auditory periphery and a loss of inhibition in subcortical and cortical brain structures, both of which are not detectable using pure-tone audiometry, have been suggested to contribute to difficulties understanding speech masked by background sound and, likely straining social interactions (Chambers, Resnik et al., 2016; Chambers, Salazar et al., 2016; Herrmann & Butler, 2021; Liberman & Kujawa, 2017; Plack et al., 2014).

Individuals with age-related hearing loss typically make behavioral adjustments—potentially unnoticed by them—before they realize that their hearing is deteriorating and seek professional help (Arlinger, 2003; Hétu & Getty, 1991; Jones et al., 1987; some may never acknowledge having hearing difficulties Hétu & Getty, 1991). For example, people with hearing loss may request that friends and family members repeat themselves or they may increase the sound level of the TV and other devices (Ranganathan et al., 2011). They may temporarily “zone out” or disengage from listening in social situations when multiple people are talking at the same time or when speech is masked by background sound, such as in busy restaurants or at birthday gatherings (Gfeller et al., 2019; Heffernan et al., 2016; see discussion also in Herrmann & Johnsrude, 2020).

Several factors make it challenging for an individual to recognize that their hearing may be impaired. Without an internal reference, many individuals fail to acknowledge a problem with hearing until social rules become violated, for example, by regularly asking others to repeat themselves (Arlinger, 2003; Weinstein & Ventry, 1982). Such behaviors may be misattributed to inattention, boredom, or rudeness, rather than hearing loss, with a negative impact on interpersonal relationships (Heffernan et al., 2016; Heine et al., 2002; Noble, 2009). Negative stigma around hearing loss may further threaten the person's self-image and may result in denial (Noble, 2009). Individuals may feign keeping up with a conversation in order to avoid revealing their hearing loss (Hétu & Getty, 1990), resulting in a lack of comprehension and unsatisfactory social interactions. The impact of hearing loss on communication and social interactions can vary depending on the exact nature of communication required in differing social contexts, for instance, in one-on-one conversations, group settings, and in more public-facing events, which place different response demands on the hearing-impaired listener. For those with severe hearing loss, even speech understanding in quiet environments or when the communication partner is turned away may pose a problem. Although these challenging situations may be just as varied as the individuals acting within them, the consequences are similar: the person with hearing loss cannot participate in social situations in the same way as they used to, or as a normally hearing person can, and they may experience strained relationships or diminished social participation as a result.

## Social Isolation in Late Adulthood

A range of terms have been used in the literature to describe reductions in the frequency, variety, and quality of social interactions (Coyle & Dugan, 2012; Mick et al., 2018; Nicholson Jr, 2009). Terms used include social withdrawal, social disconnectedness, loneliness, and social isolation (Cornwell & Waite, 2009; Mick et al., 2014; Routasalo et al., 2006; Townsend, 1979).

The terms “social withdrawal” and “social disconnectedness” overlap conceptually but are less frequently used compared to “loneliness” and “social isolation.” For example, social withdrawal has been defined as “isolating oneself from the peer group” (Rubin et al., 2011, p. 435) and social disconnectedness as “a lack of contact with others” and “a small social network, infrequent social interaction, and lack of participation in social activities and groups” (Cornwell & Waite, 2009, p. 8). “Social withdrawal” implies an active pulling away from social events, whereas “social disconnectedness” describes a state of affairs that may (or may not) have been outside of the affected individual's control.

“Loneliness” describes an individual's subjective feeling that they lack companionship (Mick et al., 2018; Shankar et al., 2013). It is often assessed through self-reported experiences or feelings, such as “How often do you feel you can find companionship when you want it?” or, “How often do you feel that you are ‘in tune’ with the people around you?” (Russell, 1996, p. 23). “Social isolation,” on the other hand, is specific to the quality and quantity of the individual's social network (Delisle, 1988) and is measured in a variety of ways, for example, by assessing an individual's social network diversity, frequency of social contacts, and social engagement Bott & Saunders, 2021; Koehn et al., 2022; Shukla et al., 2020). Loneliness and social isolation are conceptually linked, because loneliness can be experienced by an individual regardless of the breadth of their social network (Zavaleta et al., 2014). For instance, someone who is socially isolated may not feel lonely because they prefer this arrangement, whereas loneliness may be felt by someone with moderate social connections (Coyle & Duggan, 2012). These examples are consistent with the weak-to-moderate association between social isolation and loneliness measures in older adults (Coyle & Duggan, 2012).

When social isolation is assessed via self-report measures (Shukla et al., 2020), this may conflate loneliness and social isolation, as people may be reporting feelings of loneliness rather than social network size or other aspects of social participation (or lack thereof). Some authors, however, have argued for a definition of social isolation that includes subjective feelings accompanying a lack of interactions (Lien-Gieschen, 1993; Nicholson Jr, 2009), as well as societal and cultural factors such as poverty, inequality, and exclusion that produce isolation (Grenier et al., 2022; Weldrick & Grenier, 2018). More recently, a questionnaire called the Social Isolation Index has been developed to capture the multiple elements of the social isolation construct (Wister et al., 2019; but see Zavaleta et al., 2017 for an earlier review). For the upcoming sections, we use the term “social isolation” to describe the reduction in quality, types, and frequency of meaningful interpersonal relationships (Zavaleta et al., 2014), but we will later propose a vocabulary to label different facets of the condition that may relate to hearing loss in different ways.

Social isolation is common in community-dwelling older adults. Around 25% of people aged 65 years or older report feeling socially isolated (Cudjoe et al., 2020; National Academies of Sciences, Engineering, and Medicine et al., 2020; Weinstein & Ventry, 1982), with a further approximately 4% characterizing themselves as severely socially isolated. Other work suggests that more than 33% of adults aged 45 or older report feelings of loneliness (National Academies of Sciences, Engineering, and Medicine et al., 2020). Why a person becomes socially isolated may change with age (Cudjoe et al., 2020). Moreover, progression to social isolation is complex and influenced by several factors such as poor health, mobility issues, death of a partner and living alone, a lack of transportation, unemployment, or exposure to domestic or community violence (Bruce et al., 2019; Duncan Gallie & Jacobs, 2003; Tung et al., 2019).

## The Link Between Hearing Loss and Social Isolation

Several works have identified an association between age-related hearing loss and social isolation (Whitson et al., 2018; for systematic reviews see Chia et al., 2007; Pronk et al., 2011, 2014). Other studies have examined the link between hearing loss and loneliness and found that the risk of loneliness increases with increasing pure-tone audiometric hearing thresholds (Cohen-Mansfield et al., 2016; Nachtegaal et al., 2009; Sung et al., 2016). Moreover, people with severe hearing loss, defined as a threshold of 65–80 dB HL (Chadha et al., 2021; World Report on Hearing: https://www.who.int/publications/i/item/9789240020481), report substantially higher loneliness compared to people with normal hearing (typically 20 dB HL or below; Chadha et al., 2021; Sung et al., 2016; typically in the 0.5–4 kHz frequency range).

Although several researchers report a link between hearing loss and social isolation, others have failed to find this general relationship (Dawes et al., 2015; Mick et al., 2014, 2018; Pronk et al., 2011, 2014), but note associations between social isolation and hearing loss for specific subgroups. For example, greater hearing loss has been associated with social isolation in American women, but not men, aged 60–69 years (Mick et al., 2014), and greater loneliness in non-users of hearing aids, and men, in comparison to women (Pronk et al., 2013). Social lives are strongly gendered, possibly explaining low or absent correlations between hearing loss and social isolation in some subgroups. Yet other researchers have found no evidence for reduced social isolation or loneliness due to hearing loss (Christian et al., 1989; Pronk et al., 2011, 2014).

Methodological factors may contribute to discrepant findings in published work relating hearing loss to loneliness and social isolation. Some studies use remote participation to circumvent mobility issues that can affect older adults, thus enabling large sample sizes (e.g., ∼20,000; Pronk et al., 2014; Wells et al., 2020). However, data acquisition for such large numbers of participants often prohibits in-person laboratory examination, and instead relies on surveys conducted over the telephone. Telephone surveys can have low response rates, sometimes as low as 18% (Wells et al., 2020) that can lead to an unintentional selection of specific groups of people. Since these samples are more likely to capture those with existing comorbidities (e.g., mobility restrictions), this could result in observing more severe negative social impacts than are typical for the population. Other selection biases may be important, too. Older people with hearing loss may not opt to participate (Shukla et al., 2020) owing to communication challenges brought on by hearing loss, resulting in an underestimation of the impact of hearing loss on social isolation, or, perhaps, individuals who are especially troubled by hearing loss may be more enthusiastic about participation as they may want to communicate their experiences. Measurement of hearing loss has also not been consistent. Some researchers have quantified hearing loss as intelligibility of speech masked by background sound (Nachtegaal et al., 2009; Pronk et al., 2011), whereas others have used pure-tone thresholds from audiometric testing to assess hearing abilities (Sung et al., 2016; Weinstein & Ventry, 1982; Christian et al., 1989) and others have used self-reported hearing loss (Pronk et al., 2011).

**Figure 1.**
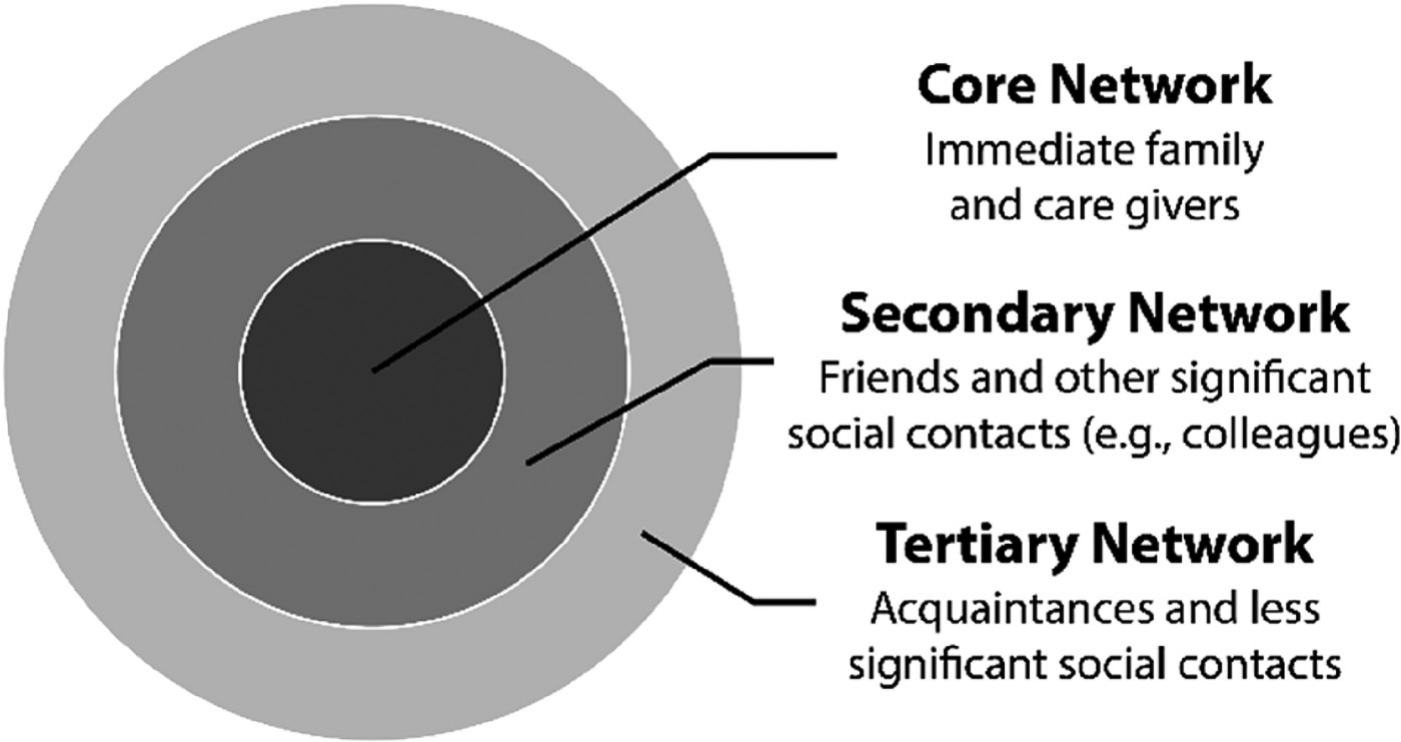
Layers of social relations. Three different layers associated with social network size have been proposed: Core network, secondary network, tertiary network (Kahn & Antonucci, 1980). Age-related vision loss appears to specifically reduce the tertiary network, but not the core and secondary networks (Wahl et al., 2013). Whether age-related hearing loss leaves core and secondary networks intact is unknown.

The extent to which specific structural aspects of a person's social networks are adversely affected by hearing loss may be another factor that possibly contributes to variations in the observed relationship between hearing loss and social isolation. According to the framework of Kahn and Antonucci (1980), social relations fall into one of three hierarchical layers depending on emotional closeness (Figure 1). The core layer includes immediate family and care givers, the secondary layer includes friends and other important contacts (e.g., coworkers, neighbors), and the tertiary layer includes acquaintances and less significant contacts (Kahn & Antonucci, 1980). Age-related vision loss appears to adversely affect social relationships in the tertiary layer, but not in the other two layers (Wahl et al., 2013). Whether age-related hearing loss also affects layers differentially is currently unclear, but highly relevant to developing interventions. Moreover, aging is associated with adaptations of social motives and goals, such that older adults may be more selective in their pursuit of social interactions and participation, focusing on positive emotional experiences in contrast to pursuing knowledge-related goals (Carstensen et al., 1999). This may mean that when they experience frequent hearing challenges, they may focus increasingly on more meaningful and higher quality social interactions (i.e., core layers; Figure 1), compared to prioritizing network size stability (i.e., secondary and tertiary layers).

The benefits of hearing-loss treatment (e.g., through provision of hearing devices) on social lives are unclear. A longitudinal study indicates that social activity levels may increase after an individual is fitted with hearing aids (Holman et al., 2021). In contrast, hearing aids provided no decrease in loneliness to those with mild and severe hearing loss (Wells et al., 2020). A 5-year longitudinal study that explored loneliness in hearing aid and cochlear implant users found no long-term improvement in loneliness after 5 years of hearing-loss treatment (Applebaum et al., 2019). A large randomized clinical trial is currently being conducted to longitudinally investigate the effects of hearing interventions on cognitively normal older adults (Sanchez et al., 2020; Deal et al., 2018). Outcome measures include quality-of-life assessments of social engagement, loneliness, depressive symptoms, and health-related quality of life. The findings from this will aid our understanding of how cognitive decline progresses with and without sensory interventions, and the contributing role of social factors (Sanchez et al., 2020). The first report from this study evaluated the effects of audiologic rehabilitation (AR), including extensive training and counseling, alongside the provision of hearing aids. There was no overall benefit on the composite measure of cognitive outcome, although a sub-group of individuals who started with poorer cognition and higher prevalence of other risk factors for dementia showed benefits in cognitive function from the intervention (Lin et al., 2023). The impact of hearing aids on social engagement has not yet been systematically evaluated in this dataset.

In sum, although substantial research is consistent with hearing loss increasing the risk for social isolation (Bott & Saunders, 2021, and see summary work of Shukla et al., 2020), some studies have found no link, possibly due to methodological and conceptual differences, or have found that negative effects of hearing loss on social engagement depend on specific demographic factors such as the gender of the respondent (Mick et al., 2014). The trajectory from diagnosis of hearing loss to social isolation is idiosyncratic and depends on a range of factors which are poorly understood, but seem to include demographic characteristics, life circumstances, comorbidities, and personality. Evidence of treatment success through assistive devices is limited so far. As described in the introduction, research has typically focused on the later stages of age-related hearing loss, when changes in social behaviors and reduced social networks may be well established and are difficult to treat.

## Psychological and Social Factors That may Influence the Relation Between Hearing Loss and Social Isolation

People with hearing impairment may employ different coping strategies (Gomez & Madey, 2001), some of which may protect more than others from social isolation. Individuals may minimize the impact of hearing loss on their social lives by actively using a hearing aid or adopting adaptive strategies, such as asking companions to keep their face visible when they are talking, or to repeat what they just said. In contrast, other individuals may disengage in demanding listening environments by momentarily zoning out; by electing not to participate in conversations when acoustic demands are high; or by avoiding challenging listening situations altogether (Demorest & Erdman, 1987; Hallberg & Carlsson, 1991; Heffernan et al., 2016; see also discussion in Herrmann & Johnsrude, 2020). These latter strategies may increase the risk of social isolation.

When a person with hearing loss has little control over the ambient acoustics, such as in public spaces or around casual acquaintances, avoidance may be a common strategy (Heine & Browning, 2004). In contrast, sound environments such as a person's own home can be controlled more easily enabling adaptive strategies such as turning off background music when people visit, or configuring the seating such that the person with hearing loss is in the center of the group.

Other factors linked to social support may exacerbate the adverse effect of hearing loss on social contacts but may not be under the direct control of the individual. For example, the loss of a spouse or the death of friends can dramatically reduce a person's social network (Farquhar, 1995; Pray, 1992; Smith, 2012). If an absent spouse or friend had been a strong motivator for an individual to participate in social activities despite experiencing hearing difficulties, such loss would be particularly detrimental.

The negative impact of hearing loss on communication is felt both by the person with hearing loss and by their interlocutor. A person with hearing loss may need to adjust how they see themselves in social contexts (Wallhagen, 2010) and what is expected of them socially (Piercy & Piercy, 2002; Wallhagen, 2010). For example, an individual with hearing loss may need to adjust to having a less prominent social role within their network because keeping up with different speakers in a social setting is more challenging (Arlinger, 2003; Heffernan et al., 2016). Those with hearing loss can feel embarrassed to regularly ask their partners to repeat speech (Piercy & Piercy, 2002; Wallhagen, 2010), while the partners are also exasperated by having to accommodate requests about repeating speech or speaking louder (Barker et al., 2017). Close partners (e.g., spouses) of individuals with hearing loss may thus also experience effort and frustration (Hétu et al., 1993; Valla & Sweetow, 2000). Partners may further limit socializing as a couple, leading to the individual with hearing loss feeling more isolated (Hétu et al., 1993; and see Scarinci et al., 2012 for third-party disability).

Finally, hearing loss and hearing aid use are often stigmatized, which may reduce self-esteem (Wallhagen, 2010). An affected individual may feel less confident, and so engage in social interactions and activities less often (David & Werner, 2016). The degree of stigma experienced may depend on gender and age; for example, hearing loss carries more stigma for younger women than for older women (Erler & Garstecki, 2002).

## Distinguishing Stages Along the Trajectory of Changes in Social Behaviors Related to Hearing Loss

**Figure 2.**
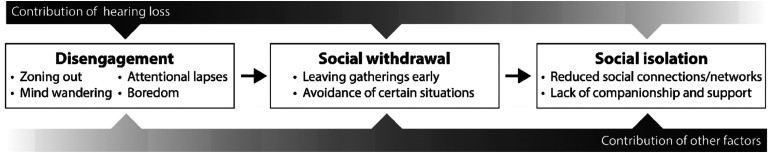
Proposed conceptual anchor stages across a trajectory of change in social behaviors, experiences, relationships, and networks associated with hearing loss. “Within-situation disengagement,” the earliest stage, refers to a tendency to temporarily zone out or mind-wander within a listening situation due to it being very challenging. “Social withdrawal,” the next stage, refers to behaviors that minimize the frequency and duration of social interactions as a way to cope with the listening challenges they impose. Finally, “social isolation” refers to a late stage when the individual's social network size is reduced after a long period of social withdrawal. The graded bars and associated arrows refer to the suggestion that hearing loss may contribute more to social behaviors at early stages (i.e., disengagement), whereas other factors, such as mobility issues, are important predictors for late-stage social isolation. A darker gray in the gradient indicates a greater contribution of hearing loss (top) or other factors such as life circumstances, comorbidities or personality (bottom), to social behaviors, experiences, relationships, and networks.

In the following section, we propose a framework comprising three conceptual “anchor stages” that capture progressive changes in social behaviors associated with hearing loss (Figure 2). We describe the initial stage as “within-situation disengagement” during which individuals “zone out” frequently and fail to participate fully in a listening situation (Gfeller et al., 2019; Heffernan et al., 2016). We call a second stage “social withdrawal” and consider it a transition period in which individuals start actively avoiding certain social situations because of their hearing loss (Arlinger, 2003; Knutson & Lansing, 1990). We refer to the third stage as “social isolation”: this is when avoidance behaviors and reduced social networks have become established (Zavaleta et al., 2014). The three stages are meant to provide descriptive anchors without implying distinct boundaries: social behaviors, relationships, and networks may change continuously over time. In practice, longitudinal trajectories that lead to social isolation are highly idiosyncratic and hearing loss is only one contributing factor. Nevertheless, this “anchor stage” framework may be useful for developing research questions and effective interventions related to the impact of hearing loss on social behaviors, experiences, and relationships. We describe the three stages in detail in the next sections and identify potential opportunities for intervention.

### Within-Situation Disengagement

Hearing loss is associated with difficulties understanding speech when it is masked by other sounds such as background music or other people talking (Johnsrude & Rodd, 2016; Schneider et al., 2000; Van Hedger & Johnsrude, 2022). Background noise renders listening effortful and fatiguing (Eckert et al., 2016; Herrmann & Johnsrude, 2020; Peelle, 2018; Pichora-Fuller et al., 1995, 2016). A person who experiences substantial effort and fatigue when listening to masked speech may mentally disengage or “zone out” for periods of time without physically withdrawing themselves from the situation (Gfeller et al., 2019; Holman et al., 2021; Heffernan et al., 2016). We refer to this process of consciously or unconsciously failing to participate fully in a listening situation as “within-situation disengagement” (see also Heffernan et al., 2016). Within-situation disengagement is a normal feature of listening, but is transient in most people with normal hearing, and does not interfere with following and enjoying a conversation. Within-situation disengagement appears to be more disruptive, and may be more frequent and longer in duration in older people with hearing loss, given the reports of this issue by people from the community (Gfeller et al., 2019; Heffernan et al., 2016). Within-situation disengagement may interfere with comprehension of speech and the enjoyment of social interactions and might thus be an initial risk marker of progression to social isolation (Figure 2).

Within-situation disengagement from listening in the context of hearing loss has received limited research attention, although two qualitative studies (Gfeller et al., 2019; Heffernan et al., 2016) and audiology blogs (e.g., ideasforears.org.uk) have pointed to this important phenomenon. Other frameworks that focus on, for example, motivation, mind wandering, and boredom, provide additional ways to study such disengagement behaviors. Although these frameworks are not directly concerned with hearing loss (but see discussion in Herrmann & Johnsrude, 2020), they provide important insights that can help us understand the negative impacts of hearing loss on listening in social situations.

Researchers increasingly recognize the critical role of a person's motivation during difficult tasks (Botvinick & Braver, 2015; Brehm & Self, 1989; Richter, 2013; Westbrook & Braver, 2013; Yee & Braver, 2018), including understanding degraded speech (Francis & Oliver, 2018; Herrmann & Johnsrude, 2020; Koelewijn et al., 2018; Peelle, 2018; Pichora-Fuller et al., 2016; Picou & Ricketts, 2014). Motivation is a core construct in psychology and refers to “the forces that drive and direct behavior” (Dweck, 2017, p. 697). The investment of cognitive resources to achieve a difficult task is mentally costly: a person is only motivated to engage if the anticipated reward outweighs the anticipated cognitive effort (Botvinick & Braver, 2015; Richter, 2013; Westbrook & Braver, 2013; Yee & Braver, 2018). For individuals with hearing loss, the mental cost associated with effortful listening may more frequently outweigh the rewards of understanding what is said, and people may mentally disengage instead.

Another relevant field of study focuses on mind wandering (Christoff et al., 2016; Smallwood & Schooler, 2006). Mind wandering refers to the (mostly unintentional) experience of thoughts moving away from the task at hand to unrelated matters (Martínez-Pérez et al., 2021; Mrazek et al., 2013). Terms such as “daydreaming” or “zoning out” among others have been used to describe mind wandering (Baer et al., 2020; Merlo et al., 2020; Smallwood et al., 2008; Smallwood & Andrews-Hanna, 2013). Studies investigating mind wandering often employ an unstimulating, boring, vigilance task in which participants see a stream of simple signals (such as a red circle on top of a stable black background) and must press a button from time to time (Lim & Dinges, 2008; Martínez-Pérez et al., 2021). During this task, participants frequently report the experience of “zoning out” or “mind wandering” (Arnau et al., 2020; Martínez-Pérez et al., 2021). The experience in such vigilance tasks may be somewhat similar to the experience of a person with hearing loss listening to masked speech that is hard or almost impossible to understand, and recent work has demonstrated increased mind wandering under listening in noisy environments versus clear speech (Steindorf et al., 2023). Attentional lapses may be common under these circumstances (Crawford, 2020), and people's thoughts may drift off to unrelated topics.

Related to the literatures on motivation and mind wandering is work that focuses on disengagement and boredom (Eastwood et al., 2012; Elpidorou, 2014; Raffaelli et al., 2018; Westgate & Wilson, 2018). Boredom has been defined as an “aversive state of wanting, but being unable, to engage in a satisfying activity” (Eastwood et al., 2012, p. 483). Boredom can be categorized into boredom proneness, which is a stable trait describing the tendency to experience boredom regardless of the specific situation, and boredom state, which characterizes situation-specific fluctuations in boredom (Chin et al., 2017; Fahlman et al., 2013; Farmer & Sundberg, 1986; Westgate & Wilson, 2018). Although boredom proneness may mediate the relation between hearing loss and social isolation (see also Crawford, 2020), boredom state is more closely related to within-situation disengagement. Boredom frameworks posit that very low and very high task challenges can lead to task disengagement and, in turn, to boredom (Raffaelli et al., 2018; Westgate & Wilson, 2018). Disengagement and boredom frameworks would posit that the high demand experienced by those with hearing loss in many social situations puts them at higher risk of disengaging from speech listening compared to a person without hearing loss. A potential consequence is that the person experiences some combination of boredom, dissatisfaction, and frustration, during social situations and does not enjoy them(Herrmann & Johnsrude, 2020; Van Tilburg & Igou, 2012).

Within-situation disengagement may be the experiential root of more pronounced changes in social behaviors observed after the onset of hearing loss. As “zoning out” or mental disengagement behaviors occur more regularly within social situations, the individual may begin active social withdrawal behaviors (Arlinger, 2003) such as not attending social events, or leaving earlier than they otherwise might, because they wish to avoid negative experiences.

Clinical interventions, including provision of hearing aids, counseling, or psychoeducation, may be most effective at this “within-situation disengagement” stage rather than when the individual is already socially isolated. At this stage, hearing loss may be a strong predictor of changes in listening behavior, with other health-related issues contributing less to this relationship compared to subsequent stages (Figure 2). Provision of hearing aids for older adults, perhaps paired with psychoeducation, risk assessment, and counseling may slow or prevent progression to the next stage—social withdrawal.

### Social Withdrawal

We define social withdrawal as a fluid transition period between within-situation disengagement and social isolation. It is characterized by active changes in social behaviors that reduce aversive experiences associated with within-situation disengagement (Figure 2; Arlinger, 2003; Knutson & Lansing, 1990). Thoughts and behaviors associated with social withdrawal may include leaving social gatherings early, avoiding bigger gatherings and those in reverberant or noisy spaces, losing spontaneity, experiencing more apprehension about social events and needing to plan these in more detail, or generally having a mindset that social interactions are not very rewarding relative to the challenges involved (Bance, 2007; Porcelli et al., 2019).

The specific thoughts and behaviors related to social withdrawal will differ among people (Andersson et al., 1996; Holman et al., 2021). Qualitative research suggests that some people use adaptive strategies that reduce speech comprehension difficulties, such as asking others to repeat themselves, without employing social withdrawal behaviors (Gomez & Madey, 2001). Other individuals employ behaviors that mitigate aversive experiences through social withdrawal (Gomez & Madey, 2001). When coming to terms with a hearing-loss diagnosis, some individuals may also experience a process of moving from avoidance of symptoms to acceptance over time (Wänström et al., 2014), which may change thoughts and behaviors related to social withdrawal.

Whether a person with hearing loss will socially withdraw depends on additional factors beyond hearing loss. Lifestyle, hobbies, and personality traits such as extraversion versus introversion may determine a person's choice to continue to participate socially despite challenges (e.g., extraversion) or withdraw from participation instead (e.g., introversion; Weinstein 1978; but see also Cox et al. 2007; Berg & Johansson 2014; Wöstmann et al., 2021 for effects of hearing loss on personality traits). Age also appears to be an important factor in the progression to social isolation (Mick & Pichora-Fuller, 2016), with unaddressed or unacknowledged hearing loss linked to an increased risk of social isolation, but only for those between 60–69 and not 70 or older (Mick & Pichora-Fuller, 2016). Moreover, aging is often accompanied by other health issues (Jolanki, 2009; Mollenkopf et al., 2007, p.195; Wrosch et al., 2007), that, in addition to hearing loss, could increase the likelihood of social withdrawal behaviors, or even lead to social withdrawal in the absence of hearing loss. For example, a person with hearing loss who also has mobility issues may find that the required effort to participate in social gatherings in places outside of their home exceeds the anticipated reward, whereas another person with similar hearing loss but without mobility issues may find the effort acceptable given the reward. Indeed, older adults with lower levels of cognitive resources than their peers are less intrinsically motivated to engage in effortful tasks (Hess, 2014; Queen & Hess, 2018).

Although hearing loss is likely a strong predictor of within-situation disengagement from speech listening (Heffernan et al., 2016), other factors may be important contributors to social withdrawal. For example, a person with hearing loss is more likely to exhibit social withdrawal behaviors if social supports are removed—for example, after the death of their spouse or a close friend.

Whether provision of hearing aids at this stage prevents social withdrawal behavior and progression to social isolation might depend on the benefit the wearer experiences, and how well they are able to use additional assistive listening devices such as personal mics and smartphone apps. It may also depend on whether hearing loss is the only contributor to social withdrawal behaviors as other health issues may themselves lead to social withdrawal. We suggest that new behavioral habits and routines start to become established at this stage and that such routines may be hard to reverse. Nevertheless, intervention is important, because preventing the shrinking of existing social networks by reducing withdrawal behaviors may be more successful than attempts to build new social networks later. Extending the size of social networks is difficult in older age (Apostolou & Keramari, 2020). In addition to treatment through provision of hearing aids, psychoeducation and counseling may provide information about the risks for social isolation and facilitate adoption of adaptive behaviors (such as proactively choosing venues with quiet background sound for meeting friends). Integrated care models, such as “social prescribing” (medical doctors prescribing nonmedical, social, activities), might also be beneficial for older adults with multiple health issues (World Health Organization, 2019).

### Social Isolation

Social withdrawal behaviors will increase the risk for social isolation, where social connections, networks, companionship, and support will have declined (Zavaleta et al., 2014). Much of the research on hearing loss and socialization has focused on this social-isolation stage (Cohen-Mansfield et al., 2016; Nachtegaal et al., 2009). Less work has focused on within-situation disengagement and social withdrawal (but see Gfeller et al., 2019; Heffernan et al., 2016). As with social withdrawal, the biggest impact of hearing loss on an individual's social interactions may be at the most peripheral, tertiary level of relationships, whereas the core level of an individual's social network, including immediate family and care givers, may be much less affected (Figure 1; Wahl et al., 2013). At this stage, individuals may consider it more adaptive to focus on quality over the quantity of social interactions (Carstensen et al., 1999).

Although research overall points to an increased risk of social isolation for individuals with hearing loss (see systematic review by Shukla et al., 2020), not all studies find this correlation (Dawes et al., 2015; Mick et al., 2014). Furthermore, the variability in social isolation explained by hearing loss seems to be small (Chen, 1994), and hearing-loss treatment may not improve social isolation (Applebaum et al., 2019; Wells et al., 2020). At this late stage in the trajectory relating hearing loss to social behaviors and networks, additional factors that determine the risk for social isolation may have a marked influence. Chronic health issues associated with a risk of social isolation, such as reduced mobility, are more common with age (Besser et al., 2018). Age-related hearing loss may be only one factor among many that are associated with social isolation. Moreover, psychological and social factors (e.g., introversion or a diminishing social network), in addition to hearing loss, may contribute to the progression from social withdrawal behaviors to social isolation.

Because social isolation is characterized by established habits and reduced social networks, interventions at this stage that focus solely on hearing loss may be ineffective. Holistic interventions that tackle multiple issues, including psychological, social, and health issues (World Health Organization, 2019) and provide a forum for socializing, education, and/or exercise (Bott & Saunders, 2021), may be required at this stage in order to foster greater social engagement and participation (Clark et al., 2021; Saunders et al., 2021). This may involve several professionals such as audiologists, general practitioners, and social-service providers, working together. A particular challenge will be to ensure that different professionals look beyond their own discipline and focus on empowering older adults to participate meaningfully in social activities. Nevertheless, evidence for effective interventions to reduce social isolation is sparse (Bott & Saunders, 2021; Findlay, 2003), which perhaps reflects the challenge of tackling social isolation at this late stage. A more systemic approach that also takes societal and cultural aspects of social isolation among older people into account (e.g., policy around public transit, age-integrated communities) may be needed to facilitate social engagement and participation (Grenier et al., 2022; Weldrick & Grenier, 2018; see also https://extranet.who.int/agefriendlyworld/age-friendly-cities-framework/).

### Summary

In this section, we discussed three conceptual anchor stages that can help understand how individuals with age-related hearing loss may become socially isolated over time. Within-situation disengagement from listening progresses to social withdrawal behaviors and eventually culminates in social isolation. Although hearing loss is probably a large contributor to experiences of within-situation disengagement, other factors such as reduced mobility and loss of friends and family probably contribute substantially to later stages of reduced social engagement. A greater understanding of the individual and situational factors that predict within-situation disengagement may help us identify who is at risk of social isolation and may benefit from intervention, and who will continue to participate in social interactions despite hearing challenges.

## Implications for Research and Future Work

The global population is aging rapidly, and the impact of age-related hearing loss on the quality of life of elderly individuals is apparent (World Health Organization, 2017). Social interactions are essential to a high quality of life (Cacioppo & Cacioppo, 2014; World Health Organization, 2017). Integrated healthcare models, psychoeducation and counseling in tandem with audiology screenings and other public health initiatives, such as encouraging social network integration and community involvement, are potentially valuable intervention strategies to mitigate the effects of age-related hearing loss on social interactions (Cattan et al., 2005; Holt-Lunstad, 2021; Santini et al., 2020; Singh et al., 2016). with the three conceptual anchor stages we propose may be useful for reducing the effects of hearing loss on social engagement in older people and may be of interest to a range of scientists and health-care providers.

The framework proposed here may facilitate the study of within-situation disengagement. As outlined above, research on boredom (Eastwood et al., 2012; Elpidorou, 2014; Westgate & Wilson, 2018), mind wandering (Smallwood & Schooler, 2006), and motivation (Carolan et al., 2022; Herrmann & Johnsrude, 2020; Mick & Pichora-Fuller, 2016; Peelle, 2018; Pichora-Fuller et al., 2016) may be relevant to understanding listening behavior. Moreover, work on the relationship between attention and speech processing (Mottonen et al., 2014; Power et al., 2012; Rimmele et al., 2015; Wild et al., 2012; Ritz et al., 2022) could be extended to develop predictive models about when a person's attentional focus may lapse and how this may affect listening engagement and speech understanding. The growing literature on listening effort could be extended to focus more explicitly on disengagement from listening that happens when required effort exceeds the available resources (Brehm & Self, 1989; Herrmann & Johnsrude, 2020; Richter et al., 2016; Wendt et al., 2018; Zekveld & Kramer, 2014; Peelle, 2018; Pichora-Fuller et al., 2016). This work may help identify early indicators of hearing difficulties and potential risk of social isolation in future.

The framework proposed here may help hearing-health care workers, such as audiologists, who are often the first point of contact after an individual (or their partner) notices an issue with hearing. The typical person with hearing difficulties may seek help from an audiologist at a relatively late stage of hearing loss progression, potentially decades after signs of speech understanding challenges first emerged (Pichora-Fuller et al., 2016; Pichora-Fuller & Levitt, 2012). At this stage, social networks of some people with hearing loss may already be diminished. Treating hearing loss is crucial at this stage, but provision of hearing aids may have only a limited impact on the individual's social networks. They will help individuals to more successfully participate in social interactions and maintain existing relationships, but may not be sufficient to help replace social connections that have already been lost. Characterizing a person's social networks would indicate whether core, secondary, or only tertiary social contacts are affected by a person's hearing loss (Figure 1), and may facilitate the development of strategies for rebuilding or replacing social connections. Longitudinal research examining factors that mediate or moderate the relationship between hearing loss and social isolation (cf. Brotman et al., 2020; Ferrer et al., 2017; Koehn et al., 2022) is needed to better understand who is at risk of becoming socially isolated.

Hearing loss is only one factor among many that can affect a person's social networks (Dawes et al., 2015; Shukla et al., 2020; World Health Organization, 2019). We expect that hearing loss will predict within-situation disengagement relatively well but may be less predictive of social isolation (Figure 2). A person with hearing loss but who is otherwise healthy and with a good social network may manage to remain socially engaged, but when additional health-related issues or life circumstances such as loss of a spouse are added, may become more at risk of social isolation. Personality probably also plays an important role in whether a person with hearing loss will become socially isolated. Facilitating good communication and social connectedness in older adulthood may thus require a holistic health-care approach that explicitly considers the impact of a variety of factors on social interactions (Grenier et al., 2022; Weldrick & Grenier, 2018). Indeed, “social prescribing” (medical doctors prescribing nonmedical, social, activities) has been employed to manage other risks related to social isolation (Howarth et al., 2020), and this could be highly beneficial for those with hearing loss as well (https://www.socialprescribing.ca/). Social prescribing also aligns with the World Health Organization's Age-friendly Cities Framework, which proposes eight domains of urban life, including community and health care, social participation, civic participation, and employment, through which barriers to the well-being and participation of older adults can be identified and mitigated (https://extranet.who.int/agefriendlyworld/age-friendly-cities-framework/).

Finally, understanding the structure of social networks of people with hearing loss, and how these change over time, may be crucial to facilitate interventions. The three layers of social networks depicted in Figure 1 (Kahn & Antonucci, 1980) may be differentially affected at different anchor stages and this requires further research. Vision impairment appears to affect mainly the most superficial network of acquaintances and thus the least significant social contacts (Wahl et al., 2013). Whether this is also the case for hearing loss is unknown. Sociometric methods (Burns & Erdley, 2011) such as social network analysis might be used to map the breadth and depth of an individual's social integration.

## Conclusions

Many older adults experience difficulty with understanding speech in everyday social situations, making social participation challenging and increasing the risk for social isolation. Here, we reviewed the literature on the relationship between hearing loss and social isolation. We identified limitations to our understanding of this relationship and propose a longitudinal framework comprising three conceptual anchor stages, beginning with within-situation disengagement from listening, and then social withdrawal, prior to social isolation. In this framework, social isolation is the end result of a long process that affords multiple points for intervention. We outlined the distinct characteristics of each stage and suggested potential interventions to mitigate negative impacts of hearing loss on social participation. This framework may be useful for scientists who are interested in identifying risk factors (other than hearing loss) for social isolation; and for health-care providers (including audiologists and family doctors) who wish to mitigate the risk of social isolation for their clients and patients with hearing impairment.
